# Factors influencing serum calcium levels and the incidence of hypocalcemia after parathyroidectomy in primary hyperparathyroidism patients

**DOI:** 10.3389/fendo.2023.1276992

**Published:** 2023-12-05

**Authors:** Yongliang Mu, Yishen Zhao, Jiannan Zhao, Qi Zhao, Yushuai Zhang, Yang Li, Jiedong Kou, Gianlorenzo Dionigi, Xuehai Bian, Hui Sun

**Affiliations:** ^1^ Department of Thyroid Surgery, China-Japan Union Hospital of Jilin University, Jilin Provincial Key Laboratory of Translational Medicine in Surgery, Jilin Provincial Engineering, Laboratory of Thyroid Disease Prevention and Treatment, Changchun, China; ^2^ National Institute for Viral Disease Control and Prevention, Chinese Center for Disease Control and Prevention, Beijing, China; ^3^ Division of General Surgery, Endocrine Surgery Section, Istituto Auxologico Italiano Istituto di Ricovero e Cura a Carattere Scientifico (IRCCS), Department of Pathophysiology and Transplantation, University of Milan, Milan, Italy

**Keywords:** primary hyperparathyroidism, parathyroidectomy, hypocalcemia, age, alkaline phosphatase

## Abstract

**Background:**

Parathyroidectomy (PTX) is an effective treatment for primary hyperparathyroidism (PHPT) patients. Postoperative hypocalcemia is a common complication after PTX. This study aimed to analyze the factors influencing serum calcium levels and the incidence of hypocalcemia after parathyroidectomy in primary hyperparathyroidism patients.

**Methods:**

The retrospective study included 270 PHPT patients treated with PTX and collected their demographic and clinical information and their laboratory indices. Factors influencing serum calcium levels and hypocalcemia after PTX in PHPT patients were analyzed using univariate and multifactorial analyses.

**Results:**

First, in patients with normal preoperative serum calcium levels (2.20–2.74 mmol/L), the higher the preoperative alkaline phosphatase and serum phosphorus levels, the lower the postoperative serum calcium levels. Furthermore, the higher the preoperative serum calcium levels and the accompanying clinical symptoms, the higher the postoperative serum calcium levels. Low preoperative serum calcium levels were shown to be a risk factor for postoperative hypocalcemia (OR=0.022), and the optimal preoperative serum calcium threshold was 2.625 mmol/L (sensitivity and specificity were 0.587 and 0.712, respectively). Second, in the mild preoperative hypercalcemia group (2.75-3.00 mmol/L), the older the patient, the higher the preoperative and postoperative serum calcium levels, the higher the postoperative serum calcium; the lower the alkaline phosphatase and calcitonin levels, the higher the postoperative serum calcium levels. On the other hand, the younger the patient was, the more likely hypocalcemia blood was (OR=0.947), with an optimal age threshold of 47.5 years (sensitivity and specificity were 0.543 and 0.754, respectively). Third, in the preoperative moderate to severe hypercalcemia group (>3.0mmol/L), patients undergoing a combined contralateral thyroidectomy and a total thyroidectomy had low postoperative serum calcium levels.

**Conclusion:**

Patients with different preoperative serum calcium levels had various factors influencing their postoperative serum calcium levels and postoperative hypocalcemia, which facilitated the assessment of their prognosis.

## 1 Introduction

Primary hyperparathyroidism (PHPT) is a disease of the parathyroid glands, in which they produce excessive amounts of parathyroid hormone (PTH). Such excess results in elevated levels of serum calcium and decreased levels of serum phosphorus. Some patients experience bone pain, arthralgia, symptoms associated with urinary tract stones, and others (1). Parathyroidectomy (PTX) is the treatment of choice for PHPT (2). Hypocalcemia is a common postoperative complication in patients with PHPT. Severe hypocalcemia (including hypoparathyroidism and bone starvation syndrome) can cause muscle spasms in the hands and feet, cardiac arrhythmias, epilepsy, and even life-threatening symptoms (3, 4). The occurrence of these symptoms is the result of a rapid decline in PTH levels after surgery, after a long period of high PTH levels in PHPT (5). Vitamin D deficiency in preoperative patients leads to an increased probability of postoperative hypocalcemia (6).

Several studies have shown that the incidence of postoperative hypocalcemia after PHPT ranges from 4.1% to 21.3% (7–9), which shows that patients are at high risk of postoperative hypocalcemia. In order to effectively prevent and timely respond to the occurrence of postoperative hypocalcemia in PHPT patients, several research institutions have conducted research and concluded that the age, the preoperative alkaline phosphatase levels, and the preoperative blood calcium levels are related to postoperative hypocalcemia (10–12). With the introduction of routine serum calcium monitoring in several countries around the world, fewer patients with clinically significant primary hyperparathyroidism and moderate to severe preoperative serum calcium levels have been identified. Furthermore, increased numbers of patients with PHPT who are asymptomatic (or less symptomatic) and have normal or mildly hypercalcemic preoperative calcium have been diagnosed (13, 14). This phenomenon indicates that the preoperative serum calcium levels may represent a different disease stage in PHPT patients. However, no studies have stratified the factors influencing the patients’ postoperative serum calcium levels and hypocalcemia according to their preoperative serum calcium levels. Therefore, in this study, we collected clinical data from patients treated with PTX at the China–Japan Union Hospital of Jilin University (Changchun, Jilin, China). We performed a stratified analysis of the factors influencing the postoperative serum calcium levels and hypocalcemia in these patients, to investigate whether they were clinical or biochemical predictors. Our aim was to estimate the probability of postoperative hypocalcemia and to provide a more timely and effective postoperative management.

## 2 Materials and methods

### 2.1 Study design

A total of 270 PHPT patients who underwent PTX at the Department of Thyroid Surgery, China–Japan Union Hospital of Jilin University (Changchun, Jilin, China), from June 2008 to October 2022 were enrolled in the present study. Participants included 157 patients who underwent parathyroidectomy and 113 patients who underwent parathyroidectomy combined with thyroidectomy (which was also combined with ipsilateral thyroidectomy, contralateral thyroidectomy, and total thyroidectomy).

### 2.2 Study indicators

In this study, the variables analyzed were sex, age, Body Mass Index (BMI), the preoperative presence or absence of clinical symptoms, the preoperative and postoperative serum calcium levels, the preoperative serum phosphorus and PTH levels, the intraoperative levels of PTH, alkaline phosphatase, and calcitonin, and the operator, surgical procedure (either non-combined thyroidectomy or combined thyroidectomy), the postoperative paraffin pathology, the number of diseased paracrine glands (either single or multiple), and the location of the diseased parathyroid glands (either superior or inferior).

The postoperative serum calcium levels were defined on the first postoperative day. The intraoperative PTH levels were defined 30 min after the intraoperative removal of the diseased parathyroid gland. Clinical symptoms included bone pain, arthralgia, urinary stones, malignancy, vomiting, and depression. Hypocalcemia was determined as a minimum postoperative serum calcium value of <2.2 mmol/L.

### 2.3 Inclusion and exclusion criteria

The inclusion criteria were the following (1): patients surgically treated for PHPT and confirmed by postoperative pathology (2), patients with complete clinical data, and (3)≥ 18 years old patients.

The exclusion criteria were as follows (1): patients with combined severe renal insufficiency (2), patients with secondary and tertiary hyperparathyroidism, and (3) patients with incomplete clinical information.

### 2.4 Grouping method

According to their preoperative serum calcium values, all patients were divided into a preoperative normal serum calcium group (A group, 2.20–2.74 mmol/L), a preoperative mild hypercalcemia group (B group, 2.75–3.00 mmol/L), and a preoperative moderate to severe hypercalcemia group (C group, serum calcium ≥3.00 mmol/L) (4, 15).

### 2.5 Calcium supplementation

On the day after surgery, patients are empirically supplemented with intravenous calcium gluconate. On the day after simple parathyroidectomy, patients received 3 g of intravenous calcium supplementation, which was adjusted on the first postoperative day, based on the intraoperative PTH and serum calcium values. The principle of calcium supplementation in patients with combined parathyroidectomy was the same; however, empirically, patients with combined thyroidectomy tended to have a more decreased parathyroid function. Therefore, the starting dosage of calcium supplementation was higher than in patients with simple parathyroidectomy. In summary, the overall calcium supplementation profile was empirically determined on the day of surgery and on the first postoperative day, being based on the intraoperative PTH and serum calcium levels. If the serum calcium levels were >2.2 mmol/L and no symptoms of hypocalcemia were diagnosed, the amount of intravenous calcium supplementation was reduced as appropriate.

### 2.6 Definitions

Our center defines the parathyroid decline rate as the difference between preoperative PTH and intraoperative PTH levels, expressed as a percentage of preoperative PTH. Intraoperative PTH levels were measured by collecting venous blood 30 min after the intraoperative removal of the diseased parathyroid glands. The parathyroid hormone levels were measured in pg/mL. Laboratory tests included the levels of serum calcium (Ca; reference range, 2.00–2.60 mmol/L), serum phosphate (P; reference range, 0.81–1.45 mmol/L), alkaline phosphatase (ALP; reference range, 30.00–120.00 IU/L), and serum calcitonin (Cal; reference range, 0.50–6.00 pg/ml).

### 2.7 Statistical methods

Statistical analysis was performed using the SPSS 27.0 software (IBM, Armonk, NY, USA). Statistics were expressed as the arithmetic mean standard deviation for continuous variables or the frequency and percentage (%) for categorical variables. The Kruskal–Wallis rank sum test was utilized to compare continuous variables, while the Pearson chi-square test was used for categorical variables. For single-factor analysis, Pearson product-difference correlation coefficients were used when all variables were continuous; chi-square coefficients were used when all variables were categorical. Spearman correlation coefficients were used when the variables were both ranked and continuous. For multifactor analysis, linear regression analysis was employed for the factors influencing the postoperative serum calcium levels. A binary logistic regression analysis was used for factors influencing postoperative hypocalcemia. A *p*<0.05 was regarded as statistically significant. Based on the binary logistic regression analysis, statistically significant variables were selected to investigate their predictive efficacy for hypocalcemia. A receiver operating characteristic curve (ROC) was established to determine the cutoff value, sensitivity, and specificity of statistically significant factors.

## 3 Results

### 3.1 Basic patient profile

In this study, patients were divided into the preoperative normal serum calcium group, preoperative mild hypercalcemia group, and the preoperative moderate to severe hypercalcemia group. There were seven patients in the preoperative severe hypercalcemia group, who were incorporated into the preoperative moderate hypercalcemia group. The preoperative mild hypercalcemia group had the highest number of patients (104 patients, 38.5%), and the preoperative moderate to severe serum calcium group had the lowest number of patients (76 patients, 28.1%). The preoperative normal hypercalcemia group had the highest number of patients with postoperative hypocalcemia (58.9%) and the lowest number of patients with preoperative clinical symptoms (37.8%). The preoperative moderate to severe hypercalcemia group had a lower percentage of patients with postoperative hypocalcemia (22.4%) than the other two groups and also the highest rate of patients with preoperative clinical symptoms (61.8%). In all three patient groups, skeletal system symptoms were the most common (22.2%) of the preoperative concomitant clinical symptoms (**Table 1**).

**Table 1:** Distribution of the number of patients with postoperative serum calcium levels, preoperative serum calcium levels, and clinical symptoms.

Variables	Grouping	Total number of people	A group	B group	C group
Total number		270	90 (33.3%)	104 (38.5%)	76 (28.1%)
Presence or absence of clinical symptoms	Number of people with postoperative hypocalcemia levels	105 (38.9%)	53 (58.9%)	35 (33.7%)	17 (22.4%)
	Number of people with normal postoperative serum calcium levels	165 (61.1%)	37 (41.1%)	69 (66.3%)	59 (77.6%)
With or without clinical symptoms before surgery	Asymptomatic Accompanying symptoms	148 (54.8%)	56 (62.2%)	63 (60.6%)	29 (38.2%)
		122 (45.2%)	34 (37.8%)	41 (39.4%)	47 (61.8%)
	Skeletal system Urinary system Digestive system Skeletal system combined with urinary system	60 (22.2%)	23 (25.6%)	20 (19.2%)	17 (22.4%)
		18 (6.7%)	2 (2.2%)	9 (8.7%)	7 (9.2%)
		14 (5.2%)	4 (4.4%)	2 (1.9%)	8 (10.5%)
		18 (6.7%)	2 (2.2%)	6 (5.8%)	10 (9.2%)

There were statistically significant differences (*p*<0.05) among the three groups concerning the preoperative and postoperative serum calcium levels, the decline rate in serum calcium levels, the preoperative serum phosphorus and alkaline phosphatase levels, the preoperative and intraoperative PTH levels, the decrease rate in PTH, and calcitonin levels (**Table 2**). The differences in demographic characteristics, preoperative clinical symptoms, and laboratory parameters among the three groups of patients required their division into groups.

**Table 2:** Basic information and statistical values of laboratory indicators for the three groups of patients.

Variables	Statistical values (mean ± SD)			*p*
	A group	B group	C group	
Age(years old)	50.32 ± 10.50	51.57 ± 11.44	52.41 ± 9.90	*p* > 0.05
BMI(kg/m^2^)	24.16 ± 3.97	23.73 ± 3.44	23.13 ± 3.21	*p* > 0.05
Preoperative Ca (mmol/L)	2.60 ± 0.13	2.86 ± 0.07	3.28 ± 0.27	*p* < 0.05
Postoperative Ca (mmol/L)	2.20 ± 0.17	2.29 ± 0.20	2.40 ± 0.29	*p* < 0.05
Serum calcium decline rate (%)	0.16 ± 0.06	0.20 ± 0.06	0.27 ± 0.10	*p* < 0.05
Preoperative P (mmol/L)	0.95 ± 0.32	0.87 ± 0.18	0.83 ± 0.20	*p* < 0.05
Preoperative ALP (IU/L)	146.12 ± 127.03	181.07 ± 302.56	339.05 ± 453.90	*p* < 0.05
Preoperative PTH (pg/ml)	374.11 ± 622.27	345.31 ± 355.42	909.36 ± 696.78	*p* < 0.05
Intraoperative PTH (pg/ml)	38.02 ± 41.06	33.97 ± 42.22	79.63 ± 81.07	*p* < 0.05
PTH decline rate (%)	0.84 ± 0.12	0.89 ± 0.08	0.90 ± 0.66	*p* < 0.05
Calcitonin (pg/ml)	0.70 ± 0.58	1.28 ± 3.65	1.36 ± 2.32	*p* < 0.05

The incidence of postoperative hypocalcemia was higher in patients with combined thyroidectomy (*p*=0.041; **Table 3**).

**Table 3:** Occurrence of postoperative hypocalcemia in patients with different procedures.

	Number of people with postoperative hypocalcemia	Number of people with postoperative normal serum calcium	*p*
Patients undergoing parathyroidectomy alone	53 (33.76%)	104 (66.24%)	0.041
Patients with combined thyroidectomy	52 (46.02%)	61 (53.98%)	

In this study, there were more female (75.9%) than male (24.1%) participants. More patients had a single-lesion parathyroidectomy (96.7%) than multiple-lesion parathyroidectomies (3.3%). Most patients (79.6%) had a parathyroid lesion located in the inferior thyroid gland. Of the patients, 41.9% had a parathyroidectomy combined with a thyroidectomy (**Table 4**).

**Table 4:** Distribution of the number of patients in different groups.

Variables	Grouping	Number of people	Percentage
Gender	Male	65	24.1%
	Female	205	75.9%
Number of diseased Parathyroid glands	Single	261	96.7%
	Many	9	3.3%
Location of diseased Parathyroid glands	Higher	55	20.4%
	Inferior	215	79.6%
Whether PTX with thyroidectomy	PTX without thyroidectomy	157	58.1%
	PTX with thyroidectomy	113	41.2%
PTX with thyroidectomy	Ipsilateral	38	14.1%
	Contralateral	31	11.5%
	Total	44	16.3%

### 3.2 Analysis of factors influencing the postoperative serum calcium levels

In this study, 14 factors were selected, which could influence the postoperative serum calcium levels in patients with primary hyperparathyroidism. These factors included the gender, age, BMI, presence of clinical symptoms before surgery, the preoperative serum calcium, serum phosphorus, and PTH levels; the intraoperative PTH, alkaline phosphatase, and calcitonin levels; the operator, the surgical procedure; and the number and location of diseased parathyroid glands. A value of 1 was assigned to male and 0 to female participants. A value of 0 was assigned to a single diseased parathyroid gland and 1 to multiple diseased parathyroid glands. A value of 1 was assigned to the superior and 2 to the inferior location of diseased parathyroid glands. A value of 1 was assigned to combined thyroidectomy and 0 to non-combined thyroidectomy. A value of 1 was assigned to the presence of preoperative clinical symptoms and 0 to their absence.

#### 3.2.1 Analysis of factors influencing the postoperative serum calcium levels in the preoperative normal serum calcium group

Correlation analysis showed that the alkaline phosphatase (r=−0.240), preoperative PTH (r=−0.318), and the preoperative serum phosphorus (r=−0.258) levels were all negatively correlated with the postoperative serum calcium levels. These parameters were combined with low postoperative serum calcium levels in patients undergoing thyroidectomy (r=−0.242), preoperative serum calcium levels (r=0.369), patients having preoperative clinical symptoms (r=0.296), and postoperative serum calcium levels, which were positively correlated (**Table 5**).

**Table 5:** Correlation analysis of factors influencing postoperative serum calcium.

	Correlation coefficient	*p*
A group		
Alkaline phosphatase	−0.240	0.022
Preoperative serum calcium	0.369	0.000
Preoperative PTH	−0.318	0.002
Preoperative serum phosphorus	−0.258	0.014
Presence or absence of clinical symptoms	0.296	0.005
Surgical procedures	−0.242	0.022
B group		
Alkaline phosphatase	−0.326	0.001
Preoperative serum calcium	0.310	0.001
Preoperative PTH	−0.234	0.017
Calcitonin	−0.204	0.038
Age	0.333	0.001
C group		
Surgical procedures	−0.340	0.003

Furthermore, statistically significant factors were selected for regression analysis. In the patient group with normal preoperative serum calcium levels, higher alkaline phosphatase and preoperative serum phosphorus levels were associated with lower postoperative serum calcium levels. Patients with high preoperative serum calcium levels and clinical symptoms were associated with higher postoperative serum calcium levels (**Table 6**). The regression model was expressed as postoperative serum calcium levels = 1.212 − 0.0004 × alkaline phosphatase levels + 0.423 × preoperative serum calcium levels − 0.102× preoperative serum phosphorus levels + 0.091× presence or absence of clinical symptoms.

**Table 6:** Regression analysis of factors influencing postoperative serum calcium.

Variables	Regression coefficient	*p*
A group		
Constants	1.212	0.000
Alkaline phosphatase	−0.0004	0.000
Preoperative serum calcium	0.423	0.001
Preoperative serum phosphorus	−0.102	0.036
Presence or absence of clinical symptoms	0.091	0.006
B group		
Age	0.005	0.004
Alkaline phosphatase	−0.0002	0.007
Preoperative serum calcium	0.831	0.001
Calcitonin	−0.010	0.040
C group		
Constants	2.488	0.000
contralateral thyroidectomy	−0.195	0.030
Total thyroidectomy	−0.240	0.013
Ipsilateral thyroidectomy	−0.154	0.154

#### 3.2.2 Analysis of factors influencing the postoperative serum calcium levels in the preoperative mild serum calcium group

Correlation analysis revealed that the alkaline phosphatase levels (r=−0.326), the preoperative levels of PTH (r=−0.234), and calcitonin (r=-0.204) were all negatively correlated with the preoperative and postoperative serum calcium levels (r=0.310) and the age (r=0.333), which were positively correlated with the postoperative serum calcium levels (**Table 5**).

Regression analysis of selected statistically significant factors revealed that older age and higher preoperative serum calcium levels were associated with higher postoperative serum calcium levels. Lower alkaline phosphatase and calcitonin levels were associated with higher postoperative serum calcium levels (**Table 6**). The regression model used was the follows: postoperative serum calcium levels = 0.831 × preoperative serum calcium levels − 0.0002 × alkaline phosphatase levels − 0.010 × calcitonin levels.

#### 3.2.3 Analysis of factors influencing the postoperative serum calcium levels in the preoperative moderate to severe serum calcium group

Correlation analysis revealed that a combined thyroidectomy was negatively associated with the postoperative serum calcium levels (r=−0.340), while other variables were not statistically significant (**Table 5**).

Regression analysis revealed that the postoperative serum calcium levels were low in patients with combined contralateral and total thyroidectomy (**Table 6**).

### 3.3 Analysis of factors influencing postoperative hypocalcemia

#### 3.3.1 Analysis of factors influencing postoperative hypocalcemia in the preoperative normal serum calcium group

A value of 1 was assigned to the occurrence of postoperative hypocalcemia and 0 to the absence of postoperative hypocalcemia. Correlation analysis showed that the preoperative serum calcium levels were negatively correlated with postoperative hypocalcemia (r=−0.221), while other variables were not statistically significant (**Table 7**).

**Table 7:** Correlation analysis of factors influencing postoperative hypocalcemia.

	Correlation coefficient	*p*
A group		
Preoperative serum calcium	−0.221	0.032
B group		
Age	−0.280	0.004
Preoperative serum calcium	−0.252	0.010
Alkaline phosphatase	0.241	0.014
C group		
Location of the parathyroid glands	4.106	0.043

**Figure 1:**
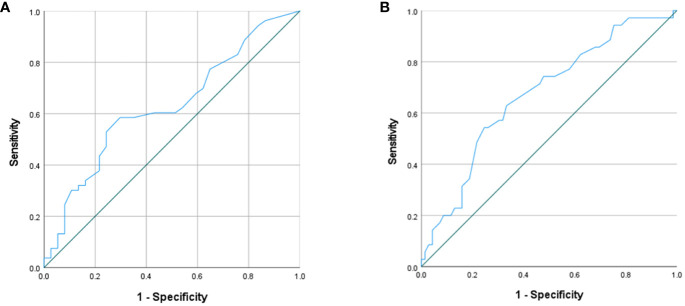
**(A)** ROC curves for preoperative serum calcium predicting postoperative hypocalcemia in the preoperative standard serum calcium group; **(B)** ROC curve for age prediction of postoperative hypocalcemia in the preoperative mildly high serum calcium group.

Regression analysis revealed that the lower the preoperative serum calcium levels, the more likely hypocalcemia would occur postoperatively (OR=0.022) (**Table 8**). ROC curves were produced to further investigate the efficacy of the preoperative serum calcium levels in predicting the occurrence of hypocalcemia in this group (**Figure 1A**). The results showed a statistically significant AUC=0.629 and *p*=0.038 at a preoperative serum calcium level of 2.625 mmol/L. The sensitivity and specificity at this point were 0.587 and 0.712, respectively (**Table 9**), which had predictive values.

**Table 8:** Regression analysis of factors influencing postoperative hypocalcemia.

Variables	Regression coefficient	*p*	OR value	95% CI
A group				
Preoperative serum calcium	−3.817	0.048	0.022	0.001–0.964
B group				
Preoperative serum calcium	−9.574	0.008	0.000	0.000–0.081
Age	−0.055	0.008	0.947	0.909–0.986
C group				
Location of the parathyroid glands	20.180	0.999	–	–

**Table 9:** ROC curve parameters.

	AUC	Cutoff values	Sensitivity	Specificity	*p*
A group					
Preoperative serum calcium	0.629	2.625	0.587	0.712	0.038
B group					
Age	0.671	47.500	0.543	0.754	0.004

#### 3.3.2 Analysis of factors influencing postoperative hypocalcemia in the mild preoperative serum calcium group

Correlation analysis showed that age (r=−0.280) and the preoperative serum calcium levels (r=−0.252) were negatively associated with the occurrence of postoperative hypocalcemia. Furthermore, alkaline phosphatase levels (r=0.241) were positively associated with the occurrence of postoperative hypocalcemia (**Table 7**).

Regression analysis revealed that age was an independent influencing factor on the occurrence of postoperative hypocalcemia (**Table 8**), being younger patients more likely to develop hypocalcemia (OR=0.947). ROC curves were produced to further investigate efficacy of age as a factor in predicting the occurrence of hypocalcemia in this group (**Figure 1B**). The results showed that when the patient’s age was 47.5 years, AUC = 0.671, *p* = 0.004, sensitivity and specificity were 0.543 and 0.754, respectively (**Table 9**), with predictive value.

#### 3.3.3 Analysis of factors influencing postoperative hypocalcemia in the preoperative moderate to severe serum calcium group

Correlation analysis revealed that the location of the diseased paracrine gland was associated with postoperative hypocalcemia; other variables were not statistically significant (**Table 7**).

The results of the regression analysis did not indicate any factors influencing the occurrence of postoperative hypocalcemia (**Table 8**).

## 4 Discussion

In this study, 270 patients were divided into three groups, according to their preoperative serum calcium levels. Statistical analysis revealed that (1) the incidence of postoperative hypocalcemia was different in each group (2), many biochemical indexes were different among the groups, and (3) the proportion of patients with or without concomitant clinical symptoms and the proportion of specific types of concomitant clinical symptoms were different among the groups. From these three observations, we conclude that the different preoperative serum calcium levels may reflect, to some extent, the stage of disease development of PHPT patients. The factors influencing the postoperative serum calcium levels and postoperative hypocalcemia in the patient groups need to be discussed. We evaluated 14 factors that could be associated with the postoperative serum calcium levels and postoperative hypocalcemia in PHPT patients. We concluded that the factors influencing the postoperative serum calcium levels and the factors influencing postoperative hypocalcemia differed among groups.

Preoperative serum calcium, serum phosphorus, and alkaline phosphatase levels affected the postoperative serum calcium levels differently, in different subgroups of patients. Possible causes were the following (1): calcium and phosphorus are the basic components of bone tissue, where both are stored as hydroxyapatite (16). Furthermore, excessive levels of PTH lead to bone destruction and to an internal environment of high serum calcium, low serum phosphorus, and increased calcitonin (17), with clinical symptoms such as bone pain and skin pruritus. Large amounts of calcium and phosphorus are required for bone remineralization after surgery (18) (2). The preoperative internal environment of high serum calcium and low serum phosphorus levels could make bone remineralization partially dependent on serum phosphorus (19). This could modify the postoperative serum calcium levels among the three groups of patients. The mean values of postoperative serum calcium levels in the three groups of patients from this study were 2.20 ± 0.17, 2.29 ± 0.20, and 2.40 ± 0.29, respectively. These values may further indicate that preoperative serum calcium and serum phosphorus levels could affect the postoperative serum calcium levels of the patients. Patients with high preoperative serum calcium levels may also maintain a relatively high serum calcium concentration postoperatively (3). The long-term bone destruction and high calcium levels, which cause some clinical symptoms in the body, also activate osteoblasts. Alkaline phosphatase is secreted by osteoblasts, which, accordingly, leads to elevated alkaline phosphatase levels, absorbing serum calcium into the bone and lowering serum calcium levels (20).

The presence or absence of clinical symptoms and calcitonin also have an impact on a patient’s postoperative calcium levels. Over the past 50 years, with the routine implementation of serum calcium monitoring, the clinical features of PHPT patients have changed from moderately severe hypercalcemia, with skeletal or renal system symptoms, to normal or mild serum calcium, with few or no symptoms (13, 14). This alteration indicates that clinical symptoms represent, to some extent, the degree of disease in patients with PHPT. As evidenced by the statistics of this study, patients with clinical symptoms have relatively high preoperative and postoperative blood calcium levels. In some institutions, calcitonin has been used to control hypercalcemia caused by PHPT, and improved results have been achieved (21, 22). This achievement may explain the effects of the levels of preoperative serum calcium, serum phosphorus (with or without clinical symptoms), and calcitonin on the postoperative levels of serum calcium. Several studies (11, 17, 23) had the same or similar results as the present study.

According to this study, we found that in the preoperative moderate to severe hypercalcemia group, patients undergoing a combined contralateral thyroidectomy and a total thyroidectomy had lower postoperative serum calcium levels than those subjected only to parathyroidectomy. Concerning the incidence of postoperative hypocalcemia, patients undergoing a combined parathyroidectomy and thyroidectomy had a higher incidence than those subjected only to parathyroidectomy (46.02% vs. 33.76%). Possible reasons for this difference could be the complex and delicate process of blood supply to the parathyroid glands and the challenge of combined thyroidectomy to achieve *in situ* preservation of the non-lesioned parathyroid glands. Moreover, the arteries and veins supplying the parathyroid glands are subjected to mechanical, electrical, and thermal injuries, which affect the blood supply to the remaining parathyroid glands (24, 25). Pradhan et al. (26) verified that combined thyroidectomy was an independent influence on postoperative hypocalcemia, which is consistent with the findings of the present study.

The younger the patients, the more likely they are to suffer from hypocalcemia. In PHPT patients, PTH overproduction leads to bone destruction and osteodystrophy (27, 28). After PTX, osteoclast activity is markedly reduced, osteoblast activity is delayed, the bone formation rate exceeds its destruction rate, calcium is transferred from the blood to the bone, and postoperative hypocalcemia occurs (29). Compared to younger individuals, older people have significantly reduced expression levels of many osteogenic genes and significantly fewer osteoblasts (30). This difference suggests that younger patients have a more evident ability to transfer calcium from the blood to the bone after PTX than older adults. Furthermore, they are more likely to develop hypocalcemia after surgery, which explains the relationship between hypocalcemia and age after PTX. Several studies have also demonstrated that patients who develop hypocalcemia tend to be younger (31, 32).

However, some studies have shown that patients with preoperative serum calcium levels >2.75 mmol/L were more likely to develop postoperative hypocalcemia than patients with normal preoperative serum calcium levels (11). The patient’s age, the preoperative serum calcium levels, and the presence or absence of clinical symptoms were not associated with the development of postoperative hypocalcemia, which contrasts with the findings of this study (33, 34). Possible causes could be the different exclusion criteria and grouping. Another study concluded that the preoperative PTH levels were associated with postoperative hypocalcemia, which has not been confirmed in the statistics of this study (35).

This study has the following shortcomings: it is a single-center retrospective study, which could have a bias in patient selection and did not exclude any potential single-center effects. The sample size was insufficient, as the number of patients in the preoperative severe serum calcium group was low and insufficient for data analysis. To assess its accuracy, the model constructed in the present study needs to be validated using additional data. Moreover, the sensitivity of the predictors was relatively low, but it can be used as a reference for all experts and scholars. Second, a long-term follow-up of the patients was not conducted in time, and the incidence of hypocalcemia in discharged patients was also not included. Therefore, larger samples, multicenter, and numerous prospective studies are needed in the future.

## 5 Conclusions

In the present study, for the first time, PHPT patients were stratified according to their initial serum calcium levels. The influencing factors of the postoperative serum calcium levels and postoperative hypocalcemia in patients with different preoperative serum calcium were analyzed. Different conclusions were obtained for different levels of preoperative serum calcium groups. Such differences are beneficial for the preoperative prognosis of the postoperative serum calcium levels and hypocalcemia in PHPT patients. They are also beneficial for a timely formulation of more accurate and individualized calcium supplementation regimens after surgery, in order to shorten the patients’ hospital stay and reduce their treatment costs.

## Ethical statement

The authors are accountable for all aspects of the work in ensuring that questions related to the accuracy or integrity of any part of the work are appropriately investigated and resolved. The study was conducted in accordance with the Declaration of Helsinki (as revised in 2013). The study was approved by the Institutional Review Board of China–Japan Union Hospital of Jilin University (approval number: 20220904014). A detailed informed consent form is signed by the patients or their legal guardians prior to surgery.

## Data availability statement

The original contributions presented in the study are included in the article/supplementary material. Further inquiries can be directed to the corresponding authors.

## Ethics statement

The studies involving humans were approved by Institutional Review Board of China-Japan Union Hospital of Jilin University. The studies were conducted in accordance with the local legislation and institutional requirements. The participants provided their written informed consent to participate in this study. The authors are accountable for all aspects of the work in ensuring that questions related to the accuracy or integrity of any part of the work are appropriately investigated and resolved. The study was conducted in accordance with the Declaration of Helsinki (as revised in 2013). The study was approved by the Institutional Review Board of China–Japan Union Hospital of Jilin University (approval number: 20220904014). A detailed informed consent form is signed by the patients or their legal guardians prior to surgery.

## Author contributions

YM: Data curation, Investigation, Methodology, Software, Writing – original draft, Writing – review & editing. YiZ: Funding acquisition, Methodology, Supervision, Writing – original draft. JZ: Formal Analysis, Methodology, Writing – review & editing. QZ: Investigation, Methodology, Writing – original draft. YuZ: Data curation, Investigation, Writing – original draft. YL: Data curation, Investigation, Writing – original draft. JK: Data curation, Investigation, Writing – original draft. GD: Methodology, Supervision, Visualization, Writing – review & editing. XB: Funding acquisition, Resources, Supervision, Validation, Writing – review & editing. HS: Conceptualization, Funding acquisition, Supervision, Visualization, Writing – review & editing.
